# Emergency Department Visits for Tick Bites — United States, January 2017–December 2019 

**DOI:** 10.15585/mmwr.mm7017a2

**Published:** 2021-04-30

**Authors:** Grace E. Marx, Melanie Spillane, Alyssa Beck, Zachary Stein, Aaron Kite Powell, Alison F. Hinckley

**Affiliations:** ^1^Division of Vector-Borne Diseases, National Center for Emerging and Zoonotic Infectious Diseases, CDC; ^2^Center for Surveillance, Epidemiology, and Laboratory Services, CDC.

The incidence of tickborne diseases in the United States is increasing; reported cases more than doubled from >22,000 in 2004 to >48,000 in 2016 (*1*). Ticks are responsible for approximately 95% of all locally acquired vectorborne diseases reported by states and the District of Columbia, with Lyme disease accounting for >80% of those cases (*2*). After a tick bite, persons might seek care at an emergency department (ED) for tick removal and to receive postexposure prophylaxis, which has been shown to effectively prevent Lyme disease when taken within 72 hours of a high-risk bite (*3*). Using data from CDC’s National Syndromic Surveillance Program (NSSP), investigators examined ED tick bite visits during January 2017–December 2019 by sex, age group, U.S. region, and seasonality. During this 36-month period, 149,364 ED tick bite visits were identified. Mean cumulative incidence was 49 ED tick bite visits per 100,000 ED visits overall; incidence was highest in the Northeast (110 per 100,000 ED visits). The seasonal distribution of ED tick bite visits was bimodal: the larger peak occurred during the spring and early summer, and the smaller peak occurred in the fall. This pattern aligns with the seasonality of a known and abundant human-biter, the blacklegged tick, *Ixodes scapularis* (*4*). Compared with other age groups, pediatric patients aged 0–9 years accounted for the highest number and incidence of ED tick bite visits; incidence was higher among male patients than among females. Tick bites are not monitored by current surveillance systems because a tick bite is an event that in and of itself is not a reportable condition to health departments. Syndromic surveillance of ED tick bite visits can provide timely information that might predict temporal and geographic risk for exposure to tickborne diseases and guide actionable public health messaging such as avoiding tick habitats, wearing repellent consistently when outdoors, and performing regular tick checks during times of increased tick bite risk.

Health care visits were identified using CDC’s NSSP BioSense Platform, which hosts a national public health surveillance system that aggregates data by U.S. Department of Health and Human Services (HHS) geographic regions.* By the end of calendar year 2019, NSSP included data from an estimated 71% of all ED visits in the United States, with 3,206 ED facilities actively contributing data.^†^ Health care visits at facilities categorized as EDs were included in this analysis; other visit categories such as inpatient hospitalizations, urgent care, or outpatient clinic visits were excluded. Data were extracted using the Electronic Surveillance System for the Early Notification of Community-based Epidemics (ESSENCE),^§^ a tool in the BioSense Platform. In collaboration with syndromic surveillance and vectorborne disease epidemiologists from states with high incidences of tickborne diseases, a query was developed to identify ED visits by patients with a chief complaint for ticks or tick bites. The query used Boolean operators (e.g., tick or tick and bite) and included common misspellings. Diagnostic codes specific to tick bites were not available in any of the diagnostic code classification systems, including the ninth and tenth revisions of the *International Classification of Diseases* and so were not included in the query.

The tick bite query was applied to all ED visits during January 1, 2017–December 31, 2019, available in ESSENCE to identify ED tick bite visits. Absolute counts and incidence of ED tick bite visits were computed by sex, age group, month, and geographic region.^¶^ Incidence was calculated by dividing the number of ED tick bite visits by the total number of ED visits in ESSENCE in that category, multiplied by 100,000. These data were also used to create a public-facing, interactive visualization tool** to allow the public to explore the data for ED tick bite visits by region, month, and basic patient demographic characteristics.

During 2017–2019, the mean annual number of ED tick bite visits was 49,788 (mean incidence = 49 per 100,000 ED visits) (Table); the mean annual number (31,340) and incidence (110 per 100,000 ED visits) were highest in the Northeast region. Males accounted for the majority (57%) of ED tick bite visits. The mean number (10,142) and incidence (86 per 100,000 ED visits) of ED visits for tick bites were highest among pediatric patients aged 0–9 years; a second peak occurred among patients aged 70–79 years (64 per 100,000 ED visits). Seasonality was bimodal, with the first and larger peak during April through July and a second smaller peak in October through November (Figure).

**TABLE:** Cumulative number and incidence of emergency department (ED) visits for tick bites, by demographic factors, region, and month — National Syndromic Surveillance Program, United States, 2017–2019

Characteristic	2017			2018			2019			Cumulative average, 2017–2019		
	No. of tick bite ED visits*	Total no. of ED visits^†^	Incidence^§^ of tick bite visits	No. of tick bite ED visits*	Total no. of ED visits^†^	Incidence^§^ of tick bite visits	No. of tick bite ED visits*	Total no. of ED visits^†^	Incidence^§^ of tick bite visits	No. of tick bite ED visits*	Total no. of ED visits^†^	Incidence^§^ of tick bite visits
**Total**	**50,158**	**90,940,257**	**55**	**44,561**	**104,527,637**	**43**	**54,645**	**110,980,103**	**49**	**49,788**	**102,149,332**	**49**
**Sex**												
Male	28,678	39,785,212	72	24,917	46,382,359	54	30,846	49,519,825	62	28,147	45,229,132	63
Female	21,480	49,777,365	43	19,644	57,805,649	34	23,799	61,273,383	39	21,641	56,285,466	39
**Age group, yrs**												
0–9	10,720	10,704,916	100	9,196	12,057,058	76	10,511	12,886,736	82	10,142	11,882,903	86
10–19	4,143	8,243,147	50	3,527	9,246,155	38	4,135	9,865,868	42	3,935	9,118,390	43
20–29	4,691	13,764,651	34	4,118	15,512,091	27	4,822	16,163,531	30	4,544	15,146,758	30
30–39	5,216	12,357,259	42	4,752	14,274,053	33	5,542	15,206,138	36	5,170	13,945,817	37
40–49	5,010	10,539,127	48	4,508	12,111,360	37	5,641	12,792,555	44	5,053	11,814,347	43
50–59	6,780	11,356,661	60	6,005	13,044,008	46	7,407	13,686,328	54	6,731	12,695,666	53
60–69	6,634	9,315,019	71	5,797	11,100,812	52	7,888	12,097,594	65	6,773	10,837,808	63
70–79	5,043	7,101,448	71	4,764	8,604,464	55	6,251	9,499,166	66	5,353	8,401,693	64
≥80	1,921	6,406,677	30	1,894	7,552,911	25	2,448	8,158,639	30	2,088	7,372,742	28
**HHS region^¶^**												
1	12,347	4,067,333	304	10,419	6,237,317	167	15,930	6,941,317	229	12,899	5,748,656	233
2	10,279	10,941,507	94	7,358	11,634,469	63	9,524	12,004,088	79	9,054	11,526,688	79
3	10,634	10,992,838	97	8,309	11,403,157	73	9,220	12,055,553	76	9,388	11,483,849	82
4	7,825	27,908,048	28	8,047	30,030,851	27	8,294	30,692,825	27	8,055	29,543,908	27
5	5,174	15,998,559	32	5,977	20,329,466	29	7,029	20,833,532	34	6,060	19,053,852	32
6	934	6,064,208	15	899	8,297,951	11	942	10,087,091	9	925	8,149,750	12
7	1,852	4,029,845	46	1,742	4,070,726	43	1,722	4,225,766	41	1,772	4,108,779	43
8	294	2,217,989	13	290	2,309,572	13	334	2,517,931	13	306	2,348,497	13
9	693	5,569,146	12	733	6,418,490	11	869	6,789,059	13	765	6,258,898	12
10	126	1,773,107	7	787	3,795,681	21	781	4,833,260	16	565	3,467,349	15
Region**												
Northeast	33,260	26,001,678	128	26,086	29,274,943	89	34,674	31,000,958	112	31,340	28,759,193	110
Midwest	7,825	20,028,404	39	8,047	24,400,192	33	8,751	25,059,298	35	8,208	23,162,631	36
Southeast	7,026	27,908,048	25	7,719	30,030,851	26	8,294	30,692,825	27	7,680	29,543,908	26
South Central	934	6,064,208	15	899	8,297,951	11	942	10,087,091	9	925	8,149,750	12
West	1,113	9,560,242	12	1,810	12,523,743	14	1,984	14,140,250	14	1,636	12,074,745	13
**Month**												
January	545	7,492,932	7	373	9,270,005	4	481	9,046,380	5	466	8,603,106	6
February	983	6,829,363	14	961	8,446,446	11	463	8,506,546	5	802	7,927,452	10
March	1,428	7,441,914	19	1,266	8,662,761	15	1,334	9,457,533	14	1,343	8,520,736	16
April	6,678	7,134,015	94	4,344	8,427,314	52	7,824	9,045,045	87	6,282	8,202,125	77
May	10,934	7,421,685	147	12,889	8,835,952	146	12,965	9,439,181	137	12,263	8,565,606	144
June	9,476	7,017,227	135	9,413	8,376,279	112	11,027	8,897,334	124	9,972	8,096,947	124
July	5,849	7,238,783	81	5,353	8,711,041	61	6,316	9,305,038	68	5,839	8,418,287	70
August	2,471	7,838,505	32	2,812	8,834,930	32	2,903	9,278,326	31	2,729	8,650,587	32
September	1,293	7,944,542	16	1,640	8,770,367	19	1,879	9,390,582	20	1,604	8,701,830	18
October	5,252	8,199,536	64	2,753	8,913,738	31	5,424	9,343,509	58	4,476	8,818,928	51
November	4,195	7,961,834	53	2,113	8,341,256	25	3,101	9,166,370	34	3,136	8,489,820	37
December	1,054	8,419,921	13	644	8,937,591	7	928	10,104,817	9	875	9,154,110	10

**Abbreviation:** HHS = U.S. Department of Health and Human Services.

* Tick ED visits were identified by the CDC Tick Bite syndrome query (https://knowledgerepository.syndromicsurveillance.org/tick-bites-centers-disease-control-and-prevention).

^†^ Totals by category might not sum to overall total counts because of missing data in some categories.

^§^ Per 100,000 total ED visits.

^¶^ HHS Region 1 (Maine, Massachusetts, New Hampshire, Rhode Island, and Vermont), HHS Region 2 (New Jersey and New York), HHS Region 3 (District of Columbia, Maryland, Pennsylvania, Virginia, and West Virginia), HHS Region 4 (Alabama, Florida, Georgia, Kentucky, Mississippi, North Carolina, South Carolina, and Tennessee), HHS Region 6 (Arkansas, Louisiana, New Mexico, and Texas), HHS Region 5 (Indiana, Illinois, Michigan, Minnesota, Ohio, and Wisconsin), HHS Region 7 (Iowa, Kansas, Missouri, and Nebraska), HHS Region 8 (Colorado, Montana, North Dakota, and Utah), HHS Region 9 (Arizona, California, and Nevada), and HHS Region 10 (Alaska, Idaho, Oregon, and Washington).

** The *Northeast* region includes HHS Region 1 (Maine, Massachusetts, New Hampshire, Rhode Island, and Vermont), HHS Region 2 (New Jersey and New York), and HHS Region 3 (District of Columbia, Maryland, Pennsylvania, Virginia, and West Virginia); the *Southeast* region includes HHS Region 4 (Alabama, Florida, Georgia, Kentucky, Mississippi, North Carolina, South Carolina, and Tennessee); the *South Central* region includes HHS Region 6 (Arkansas, Louisiana, New Mexico, and Texas); the *Midwest* region includes HHS Region 5 (Indiana, Illinois, Michigan, Minnesota, Ohio, and Wisconsin) and HHS Region 7 (Iowa, Kansas, Missouri, and Nebraska); the *West* region includes HHS Region 8 (Colorado, Montana, North Dakota, and Utah), HHS Region 9 (Arizona, California, and Nevada), and HHS Region 10 (Alaska, Idaho, Oregon, and Washington).

**FIGURE:**
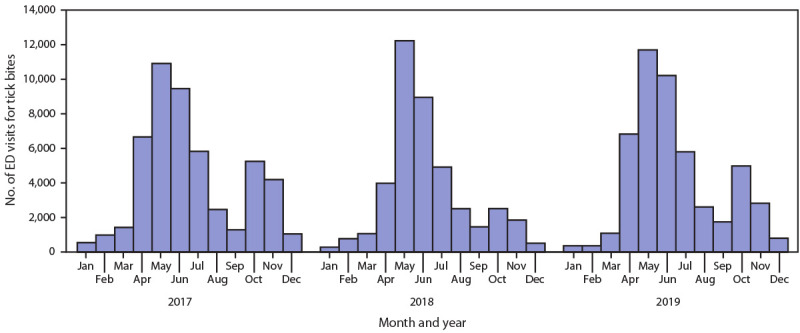
Emergency department (ED) visits for tick bites, by month — National Syndromic Surveillance Program, United States, 2017–2019

## Discussion

Syndromic surveillance using NSSP data indicates high numbers and incidence of ED tick bite visits in the United States particularly during the late spring and early summer months, when nymphal blacklegged ticks are most active (*4*). The number and rate of ED tick bite visits were highest in the Northeast, where Lyme disease is highly endemic and where tickborne disease risk might be well recognized (*5*). Male patients, as well as very young (aged <10 years) and older patients (aged 50–79 years) were most likely to seek care at an ED for tick bites.

This analysis demonstrates that many patients are sufficiently concerned about tickborne diseases to seek care at an ED after a tick bite. However, ED visits likely represent only a fraction of the total health care impact of patients seeking care after a tick bite; a study in the United Kingdom showed that ED visits accounted only for approximately 12% of all health care visits by patients for arthropod bites, with most patients (67%) seeking care at outpatient clinics (*6*). The bimodal seasonal distribution of ED tick bite visits is consistent with a New Hampshire study of ED encounters for Lyme disease (*7*). In a prospective study, tick encounters were a strong predictor of tickborne diseases in the northeastern United States (*8*). Findings from the current study closely parallel patterns seen in Lyme disease surveillance (*5*) that show that Lyme disease is reported more frequently among males and among very young and older persons, supporting the application of syndromic surveillance for tick bites as a harbinger for tickborne disease.

Syndromic surveillance represents the only national system currently available to track tick bites in humans and is a powerful complementary tool to traditional surveillance for tickborne diseases, particularly in areas with high incidence of Lyme disease, the most common U.S. tickborne disease. A major benefit of syndromic surveillance is its timeliness because most data are available within days of the health care visit. These data can guide actionable public health messaging. Tickborne disease prevention practices include avoiding tick habitats, wearing repellent consistently when outdoors, and performing regular tick checks during times of increased tick bite risk. After a high-risk tick bite, a timely single dose of doxycycline might be effective in preventing Lyme disease and is considered safe for all ages, including pediatric and geriatric populations.^††^ Another benefit of syndromic surveillance is its efficiency; because it relies on automated systems, it represents a lower cost in fiscal and human resources.

The findings in this report are subject to at least four limitations. First, the geographic granularity of these data is limited to HHS regions, which can comprise states and territories with heterogenous risks for tick exposure, ED data-sharing coverage with NSSP, and health care–seeking behavior. Given that most ED tick bite visits occurred in the Northeast, these trends might reflect primarily patient health care–seeking behavior in areas where Lyme disease is a major concern. County or state level data would reveal a more precise picture of tick bite risk and might be more informative for local public health action. Second, the query was limited to select combinations of words in patients’ chief complaints and did not include any specific diagnostic or laboratory test codes. This might have led to misclassification that could have under- or overestimated the actual impact of ED tick bite visits. Medical record reviews of ED visits identified by the query could more thoroughly characterize this surveillance system by evaluating the sensitivity, specificity, and negative and positive predictive value of the syndromic surveillance query. Third, this analysis was limited to patients seeking care at an ED and does not represent all health care visits by patients seeking care after tick bites. The analysis was restricted to ED data because data available in NSSP are most complete for ED visits. Patients who are young, single, and employed might be more likely to visit an ED than an outpatient clinic (*9*) and might be overrepresented in this analysis. Finally, this analysis is based only on data from facilities that participate in NSSP and therefore is not generalizable to patients at nonparticipating facilities.

Syndromic surveillance for tick bites is valuable as a novel and efficient method to understand past trends and current risk for tick bites by region. By accessing these data through CDC’s tick bite data tracker, a public-facing dashboard (https://www.cdc.gov/ticks/tickedvisits/index.html), public health practitioners and communities have access to immediately actionable data to guide public health messaging and individual tick bite prevention efforts (e.g., avoiding tick habitats, wearing repellent consistently when outdoors, and performing regular tick checks during times of increased tick bite risk). Educational campaigns that provide information to the public about how to safely remove ticks at home and when prophylactic antibiotics are indicated might be beneficial to reduce the impact on health care, associated health care costs, and personal risk for exposure to tickborne diseases.^§§^

**Summary**

**What is already known about this topic?**

Tickborne diseases are spread by the bites of infected ticks; approximately 50,000 cases of tickborne diseases are reported in the United States each year. National surveillance for tick bites is not currently available.

**What is added by this report?**

A novel query of National Syndromic Surveillance Program data indicated that one out of every 2,000 emergency department visits are for tick bites, with higher incidence during the spring and early summer and in the Northeast.

**What are the implications for public health practice?**

Syndromic surveillance data for tick bites can guide timely, actionable public health messaging such as avoiding tick habitats, wearing repellent consistently when outdoors, and performing regular tick checks during times of increased tick bite risk.

## Footnotes

*
https://www.hhs.gov/about/agencies/iea/regional-offices/index.html

†
https://www.cdc.gov/nssp/overview.html

§ ESSENCE is a secure, integrated web-based application that allows application of custom and standardized analytic queries to identify, evaluate, share, and store syndromic surveillance data.

¶ The *Northeast* region includes HHS Region 1 (Maine, Massachusetts, New Hampshire, Rhode Island, and Vermont), HHS Region 2 (New Jersey and New York), and HHS Region 3 (District of Columbia, Maryland, Pennsylvania, Virginia, and West Virginia); the *Southeast* region includes HHS Region 4 (Alabama, Florida, Georgia, Kentucky, Mississippi, North Carolina, South Carolina, and Tennessee); the *South Central* region includes HHS Region 6 (Arkansas, Louisiana, New Mexico, and Texas); the *Midwest* region includes HHS Region 5 (Indiana, Illinois, Michigan, Minnesota, Ohio, and Wisconsin) and HHS Region 7 (Iowa, Kansas, Missouri, and Nebraska); the *West* region includes HHS Region 8 (Colorado, Montana, North Dakota, and Utah), HHS Region 9 (Arizona, California, and Nevada), and HHS Region 10 (Alaska, Idaho, Oregon, and Washington).

**
https://www.cdc.gov/ticks/tickedvisits/index.html

††
https://www.cdc.gov/lyme/resources/FS-Guidance-for-Clinicians-Patients-after-TickBite-508.pdf

§§
https://www.cdc.gov/ticks/removing_a_tick.html
